# Retraction: *ALDH2* and *ADH1* Genetic Polymorphisms May Contribute to the Risk of Gastric Cancer: A Meta-Analysis

**DOI:** 10.1371/journal.pone.0270613

**Published:** 2022-06-23

**Authors:** The PLOS ONE Editors

Following the publication of this article [1], the corresponding author informed the journal that a third-party company handled the submission and peer review process.

Upon review of the submission history for the manuscript, the *PLOS ONE* editors found strong indications that the peer review process was compromised.

The article [1] was assessed by a member of the Editorial Board who was not involved in the original peer review process. They identified the following methodological errors that undermine the results and conclusions:

- Odds Ratio (OR) crude estimates could not be replicated from single studies included in the meta-analyses, and the use of crude estimates may have led to overestimated ORs as the role of other potential risk factors was ruled out.
- There is no statistical test named “Begger’s funnel plot.”
- The use of Egger’s test to assess potential publication bias via funnel plot asymmetry cannot be considered reliable given the number of studies included in the meta-analysis.

The Academic Editor’s assessment supports the concern that the review process was not adequate.

The authors did not respond to requests to comment on the above concerns.

In light of these concerns, the *PLOS ONE* Editors retract this article. We regret that the issues in this article were not identified prior to publication.

In response to this decision, all authors either did not respond directly or could not be reached.
